# Plasma extracellular vesicle miRNAs as potential biomarkers of superstimulatory response in cattle

**DOI:** 10.1038/s41598-020-76152-9

**Published:** 2020-11-05

**Authors:** Ahmed Gad, José María Sánchez, John A. Browne, Lucie Nemcova, Jozef Laurincik, Radek Prochazka, Pat Lonergan

**Affiliations:** 1 Institute of Animal Physiology and Genetics, Czech Academy of Sciences, Liběchov, Czech Republic; 2 Department of Animal Production, Faculty of Agriculture, Cairo University, Giza, Egypt; 3 School of Agriculture and Food Science, University College Dublin, Dublin, Ireland; 4 Constantine the Philosopher University in Nitra, Nitra, Slovakia

**Keywords:** Biotechnology, Developmental biology, Molecular biology, Physiology

## Abstract

The ability to predict superstimulatory response would be a beneficial tool in assisted reproduction. Using small RNAseq technology, we profiled extracellular vesicle microRNA (EV-miRNA) abundance in the blood plasma of heifers exhibiting variable responses to superstimulation. Estrous synchronized crossbred beef heifers (n = 25) were superstimulated and blood samples were collected from each heifer on Day 7 of consecutive unstimulated (U) and superstimulated (S) cycles. A subset of high (H) and low (L) responders was selected depending on their response to superstimulation and EV-miRNA profiles were analysed at both time-points in each heifer. Approximately 200 known miRNAs were detected in each sample with 144 commonly detected in all samples. A total of 12 and 14 miRNAs were dysregulated in UH *vs.* UL and in SH *vs.* SL heifers, respectively. Interestingly, miR-206 and miR-6517 exhibited the same differential expression pattern in H compared to L heifers both before and after superstimulation. Pathway analysis indicated that circadian rhythm and signaling pathways were among the top pathways enriched with genes targeted by dysregulated miRNAs in H *vs.* L responding heifers. In conclusion, heifers with divergent ovarian responses exhibited differential expression of plasma EV-miRNAs which may be used as a potential biomarker to predict superstimulation response.

## Introduction

Unlike high genetic merit sires, which produce billions of fertile sperm at each ejaculation and which can produce thousands of offspring during (and even after) their lifetime, the contribution of genetically superior cows is limited by the fact that they typically ovulate only one oocyte during each estrous cycle and that pregnancy lasts 9 months following which a period of uterine involution is required before any subsequent pregnancy. Thus, under normal circumstances, most cows in commercial herds have fewer than 5–10 calves in their lifetime. Induction of multiple ovulations (often termed ‘superstimulation’ or ‘superovulation’) coupled with artificial insemination (AI), embryo recovery and embryo transfer (ET), provides an opportunity to substantially increase the impact of superior females on a breeding program by allowing the gestation to occur in a surrogate recipient^1^.

Despite the fact that much research has focused on methods to increase the number of ovulations and fertilized oocytes from the donor female, the mean yield of transferable embryos produced per superovulatory cycle (6 to 8) has not changed markedly during the last 50 years^2^. Variability in superovulatory response is one of the main limiting factors affecting the success of ET technology in genetic improvement programs. Donors are normally selected on the basis of genetic merit, but the use of a reliable tool in commercial practice that would predict response to a superstimulatory treatment and embryo yield would be of great benefit to the cattle industry.

Female mammals are born with a highly variable number of follicles and oocytes in their ovaries, the so-called ‘ovarian reserve’. This reserve is established during fetal development, is not replenished post-natally and decreases with age^3,4^. The association between the ovarian reserve and fertility in female cattle has recently received attention due to the validation of two reliable markers of the size of the ovarian reserve: (i) the number of follicles recruited during follicular waves (antral follicle count, AFC)^5,6^ and (ii) peripheral concentrations of anti-Müllerian hormone (AMH), a dimeric glycoprotein and a member of the transforming growth factor β (TGF-β) family of growth and differentiation factors which is produced by the granulosa cells of pre-antral or early-antral follicles^7–9^. Using ultrasonography, the peak number of follicles recruited per wave has been shown to be highly variable among individuals but highly repeatable within animal such that cattle can be reliably phenotyped based on AFC^6^. Similarly, growing evidence indicates that AMH concentrations vary minimally during estrous cycles in cattle, implying that AMH concentrations can be reliably determined with a single blood sample on a random day of the cycle^10^.

MicroRNAs (miRNAs), a class of small non-coding RNAs that regulate gene expression post-transcriptionally, represent another potential marker of superovulatory response. A variety of miRNAs have been detected in the extracellular environment and in almost all biological fluids (reviewed by Yáñez-Mó et al.^11^). These miRNAs exist in a stable protective form associated with high-density lipoproteins^12^, Argonaute (Ago2) protein^13^, or within extracellular vesicles (EVs), particularly exosomes and microvesicles. EVs protect miRNAs and enable their transportation between different cells and tissues as mediators of intracellular communication^14,15^. These features make EV-miRNAs a potentially powerful tool in the assessment of the functional status of various cells and tissues and render them a better source of miRNAs to be used as biomarkers compared to other sources^16^. Detection of EV-miRNAs in body fluids, including blood plasma and follicular fluid, represents a non-invasive method that could reflect both physiological and pathological conditions associated with various reproductive functions^17^. Amongst different mammalian species, numerous EV-miRNAs exhibit distinct expression patterns in association with various reproductive processes including follicle and oocyte development^18,19^, oocyte fertilization and embryo quality^20,21^, oviduct function^22^, stages of the estrous cycle^23^, and embryo-maternal interaction^24,25^.

In cattle, Noferesti et al.^26^ reported that superstimulation induced changes in the expression of extracellular miRNAs in plasma and follicular fluid, most of which were closely related to ovarian function and oocyte meiosis. However, two independent groups of heifers were used (one group unstimulated, the other superstimulated), no data on superovulatory response were provided and, as animals were not inseminated, no data on embryo yield were available. Therefore, in order to identify potential markers of superovulatory response and yield of transferable embryos, the aim of this study was to characterize the plasma EV-miRNA profiles of the same heifers in an unstimulated (U) and stimulated (S) cycle. We hypothesized that heifers with a divergent ovarian response (high or low) to superstimulation treatment would exhibit differences in the abundance of EV-miRNAs in blood plasma. These differences in expression could be used as potential markers to predict the superovulatory response and aid in the selection of donors.

## Material and methods

All experimental procedures involving animals were approved by the Animal Research Ethics Committee of University College Dublin, were authorized by the Health Products Regulatory Authority under the European Union (Protection of Animal Use for Scientific Purposes) Regulations 2012 (S.I. No. 543 of 2012) as amended, and Directive 2010/63/EU of the European Parliament and were performed in accordance with relevant guidelines and regulations.

### Synchronization and superstimulation protocols

**Figure 1:**
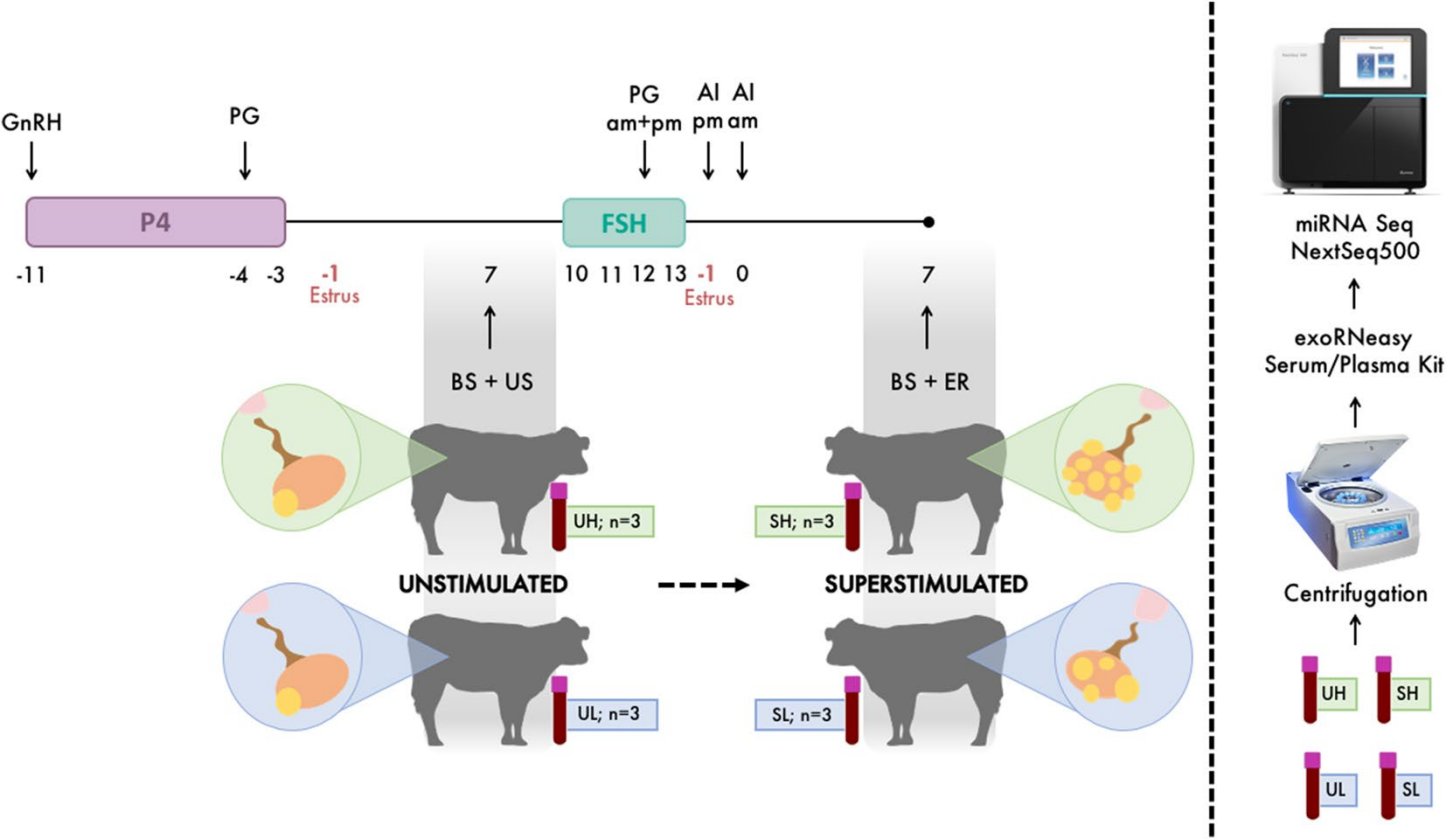
Experimental design. P4, progesterone releasing device; GnRH, gonadotrophin-releasing hormone; PG, prostaglandin F2α; BS, blood sample; US, ultrasound scanning; FSH, follicle-stimulating hormone, AI, artificial insemination; ER, embryo recovery; U, unstimulated estrous cycle; S, superstimulated estrous cycle; H, high ovulatory response in terms of the number of corpora lutea; L, low ovulatory response. Numbers indicate days relative to ovulation (Day 0), approximately 28 h after first standing to be mounted.

Samples were collected from Charolais- and Limousin-cross heifers aged between 21 and 32 months, weighing between 555 and 670 kg, and fed a diet consisting of grass and maize silage supplemented with a standard beef ration. All heifers were kept under identical farm conditions during the study. The estrous cycles of all heifers were synchronized using an 8-day intravaginal progesterone (P4) device (PRID E, 1.55 g P4, Ceva Santé Animale, Libourne, France). On the day of the PRID E insertion, each heifer received a 2 mL intramuscular (i.m.) injection of synthetic gonadotrophin-releasing hormone (Ovarelin, Ceva Santé Animale, equivalent to 100 µg Gonadorelin). One day prior to PRID E removal, all heifers received a 5 mL i.m. injection of prostaglandin F2α (Enzaprost, Ceva Santé Animale, equivalent to 25 mg Dinoprost). Only heifers displaying standing estrus were used. On Day 10 of the subsequent estrous cycle (estrus = Day -1), heifers (n = 25) were superstimulated by the administration of decreasing doses of follicle-stimulating hormone (FSH) twice a day for 4 days (455 IU of FSH in total; Folltropin, Bioniche, Bellesville, Canada) together with two injections of prostaglandin F2α (Enzaprost) separated 12 h apart on the third day of FSH treatment, and followed by AI with frozen-thawed semen 24 (Day -1) and 36 h after the last FSH injection, as described previously^27^. Day 0 was considered the day of ovulation (approximately 28 h after first standing to be mounted^28,29^) in both the U and S estrous cycle (Fig. 1).

### Corpus luteum counting and measurements and embryo recovery

Superstimulated heifers were slaughtered in a commercial abattoir 7.5 days after the first AI. Reproductive tracts were recovered and transported to the laboratory within 2 h of slaughter. All corpora lutea (CL) were dissected from the ovarian tissue, counted and weighed. Each uterine horn was gently flushed with 20 mL of pre-warmed PBS (38.8 °C) containing 5% fetal calf serum and the total numbers of recovered and transferable embryos were recorded for each heifer.

### Blood sample collection

Blood samples were collected from the coccygeal vessels of each heifer into non-heparinised tubes (EDTA BD Vacutainer, Vaud, Switzerland) on Day 7 of both the U and S estrous cycles and placed on ice. Immediately after collection, blood samples were centrifuged at 1,900×g and 4 °C for 10 min. The supernatant was further centrifuged at 16,000 × g and 4 °C for 10 min before freezing and storing at − 20 °C in plastic pour off tubes until analysis.

### EV-RNA isolation, library preparation and sequencing

**Figure 2:**
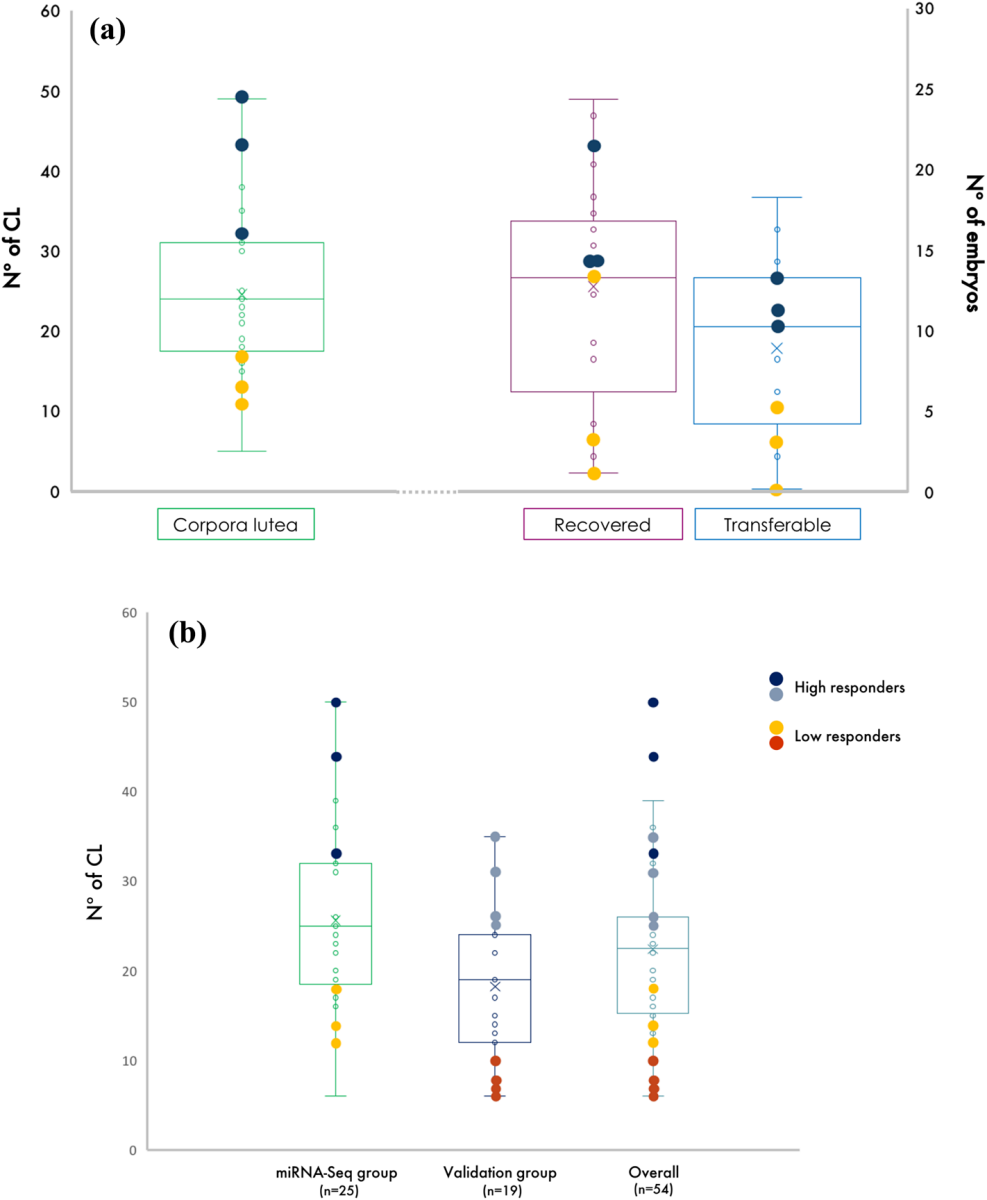
(**a**) Box-plots showing the variation in the number of corpora lutea (Nº of CL; green) and the number of recovered (purple) and transferable (blue) embryos recorded on Day 7 of the estrous cycle following superovulation (n = 25 heifers). Of note, the three dark blue and three yellow spots in each box-plot represent the three selected high and low responding heifers subjected to further analysis, respectively. (**b**) Box-plots showing the number of corpora lutea (Nº of CL) in: (i) the miRNA-Seq group (n = 25 heifers), (ii) the validation group (n = 19 heifers), and the overall (n = 44 heifers). Of note, the four grey and four red spots in each box-plot represent the four selected high and low responding heifers used for validation of miRNA-Seq data, respectively.

A subset of high (H, upper quartile, n = 3) and low (L, lower quartile, n = 3) responding heifers was selected depending on the ovarian response to superstimulation (≥ 32 and ≤ 17 CL, respectively) for EV-RNA isolation (Fig. 2a). Total EV-RNA, including miRNA, was isolated from blood plasma samples of UH, SH, UL, and SL heifers using the exoRNeasy Serum/Plasma Kit (Qiagen, Hilden, Germany) according to the manufacturer’s instructions. The RNA concentration and size distribution were analyzed on an Agilent 2100 Bioanalyzer using the Agilent RNA 6000 Pico kit (Agilent Technologies, Santa Clara, CA, USA). Furthermore, samples were checked for potential haemolysis by determining the miR ratio of miR-451a to miR-23a-3p as described previously^30^. A total of 50 ng RNA was converted into miRNA NGS libraries using the QIAseq miRNA Library Kit (Qiagen) according to the manufacturer’s instructions. Briefly, adapters containing Unique Molecular Index (UMI) were ligated to the RNA, which was then reverse transcribed into cDNA using primers containing an integrated UMI. The cDNA was amplified using PCR (22 cycles), indices were added during the PCR amplification, and then the samples were purified. Library preparation QC was performed using the Bioanalyzer 2100 (Agilent Technologies). Based on the quality of the inserts and the concentration measurements, the libraries were pooled in equimolar ratios and quantified using qPCR. The library pool was then sequenced on a NextSeq500 sequencing instrument (Illumina, Inc., San Diego, CA, USA) with a read length of 75 bases in a single-end read. Raw data were de-multiplexed and FASTQ files for each sample were generated using the bcl2fastq software (Illumina Inc.). FASTQ data were checked using the FastQC tool version 0.11.7. Average read quality score above 30 was considered as high quality.

### Sequencing data analysis

Adapter and UMI information in raw reads was extracted using Cutadapt1.11^31^, and the output was used to remove adapter sequences and to collapse reads by UMI. The raw FASTQ files and processed files have been deposited in NCBI’s Gene Expression Omnibus (GEO) and are accessible through GEO Series accession number (GSE148225). Sequence reads were mapped to the *Bos taurus* reference genome (UMD3.1) using Bowtie2 (2.2.2)^32^. Reads that had a perfect match to the reference sequences were used for annotation against miRNAs of bovine and all other organisms listed in the mirBase database (release 21). The trimmed mean of M-values method (TMM normalization) was used for normalizing the data^33^ and differential expression analysis was performed using the EdgeR statistical software package (Bioconductor, https://bioconductor.org/). MiRNAs with log2 fold change ≥ 1 and *P*-value ≤ 0.05 were considered as significantly differentially expressed (DE). The putative miRNAs were predicted from the unannotated sequences using Qiagen specific scripts based on the MiRPara tool^34^.

### MiRNA-target gene prediction and ontological classification

Differentially expressed miRNA-target genes were identified using the TargetScan analysis tool (release 7.2, https://www.targetscan.org/). A cumulative weighted context++ score ≤ − 0.2 was set as the threshold^35^. The list of predicted target genes of up- or down-regulated miRNAs for each comparison were submitted to the DAVID bioinformatics web-tool (https://david.abcc.ncifcrf.gov/) for ontological classification. Significant pathways were identified from the Kyoto Encyclopaedia of Genes and Genomes (KEGG) database^36^. Interaction networks of the targeted genes and the identified pathways were constructed by Cytoscape (https://www.cytoscape.org/) and ClueGO (https://apps.cytoscape.org/apps/cluego) ^37,38^. In addition, experimentally validated bovine miRNA-target gene interactions were obtained from the miRTarBase 8.0 database^39^ and the validated targets for the DE-miRNAs were extracted.

### Validation experiment

In order to validate the findings, an independent group of Charolais- and Limousin-cross heifers (n = 19; 16–32 months, 550–640 kg) were superovulated as described above. High (n = 4) and low (n = 4) responding heifers (Fig. 2b) were selected for validation. Blood samples were collected on Day 7 of both the U and S estrous cycles and processed for EV-RNA isolation as described above. Eight DE-miRNAs (let7-c, miR-196a, miR-139, miR-199a-3p, miR-205, miR-17-5p, miR-206, and miR-6517) were selected based on their read counts in the different group comparisons and then quantified using TaqMan miRNA assays (Applied Biosystems, Foster City, CA, USA). Briefly, 10 ng total RNA were reverse transcribed using TaqMan MicroRNA Reverse Transcription Kit (Thermo Fisher Scientific, Waltham, MA, USA) according to the manufacturer’s instruction. Quantitative PCR was performed in a RotorGene 3000 cycler (Corbett Research, Mortlake, New South Wales, Australia) using the QIAGEN OneStep RT-PCR Kit (Qiagen) in a 10 µl reaction mixture containing 2 µl 5 × reaction buffer, 0.4 µl dNTP mix (10 nM stock of each), 2 µl miRNA specific TaqMan probe, forward and reverse primer mixture (5 × stock), 0.4 µl enzyme mix, 2 µl cDNA, and nuclease-free water. Reaction conditions were as follows: initial denaturation at 95 °C for 15 min, followed by PCR cycles consisting of denaturation at 94 °C for 15 s, annealing at 60 °C for 15 s and extension at 72 °C for 15 s. Fluorescence data were acquired at the end of each extension step. Non-template and negative controls were included in all qPCR runs to validate that primers were not amplifying contaminating DNA. Data were processed using internal comparative analysis software (Corbett Research) and normalized to miR-26 expression, one of the most stably expressed miRNAs across all the samples as analysed by NormFinder software. For statistical analysis, data were analyzed using one-way ANOVA followed by multiple pair-wise comparisons using the Tukey test and the statistical significance level was defined at *P* < 0.05.

## Results

### Corpus luteum and embryo assessment

Overall, there was a large variation in the response to ovarian superstimulation with a range of 5 to 49 CL. The embryo recovery rate was also very variable, ranging from 1 to 24 and from 0 to 18 for the total number of recovered and transferable embryos, respectively (Fig. 2a). Of the 25 heifers that were subjected to superstimulation, six heifers exhibiting high (upper quartile, n = 3) or low (lower quartile, n = 3) ovulatory response based on the number of CL present on Day 7 of the S cycle (≥ 32 and ≤ 17 CL, respectively) were selected for further analysis (Fig. 2a). The number of CL, total luteal tissue weight and the number of recovered and transferable embryos recovered on Day 7 of the S estrous cycle from each of those heifers are summarized in Table 1. Briefly, mean (±SD) number of CL (13.6±3.0 *vs.* 41.3±8.6), mean (±SD) total weight of luteal tissue (33.3±13.9 *vs.* 10,713±.9 g) and mean (±SD) total number of recovered (6±6.2 *vs.* 16.3±4) and transferable (2.6±2.5 *vs.* 11.3±1.5) embryos were lower in SL *vs.* SH heifers, respectively.

**Table 1:** Summary data showing the number of corpora lutea (Nº of CL), total luteal tissue weight and the number of recovered and transferable embryos recorded on Day 7 of the superovulated cycle. Each heifer (numbered 1–3 for each group) showing a low or high response to treatment is represented as SL or SH, respectively. Overall results in each group are expressed as the mean ± standard deviation.

Treatment Group	Nº of CL	Luteal tissue weight (g)	Nº of recovered embryos	Nº of transferable embryos
SL1	11	17.3	1	0
SL2	13	42.4	4	3
SL3	17	40.3	13	5
Overall SL	13.6 ± 3.0	33.3 ± 13.9	6.0 ± 6.2	2.6 ± 2.5
SH1	32	103.4	14	11
SH2	43	95.3	21	13
SH3	49	122.4	14	10
Overall SH	41.3 ± 8.6	107.0 ± 13.91	16.3 ± 4.0	11.3± 1.5

### MiRNA sequencing data and global detection of EV enclosed miRNAs

**Figure 3:**
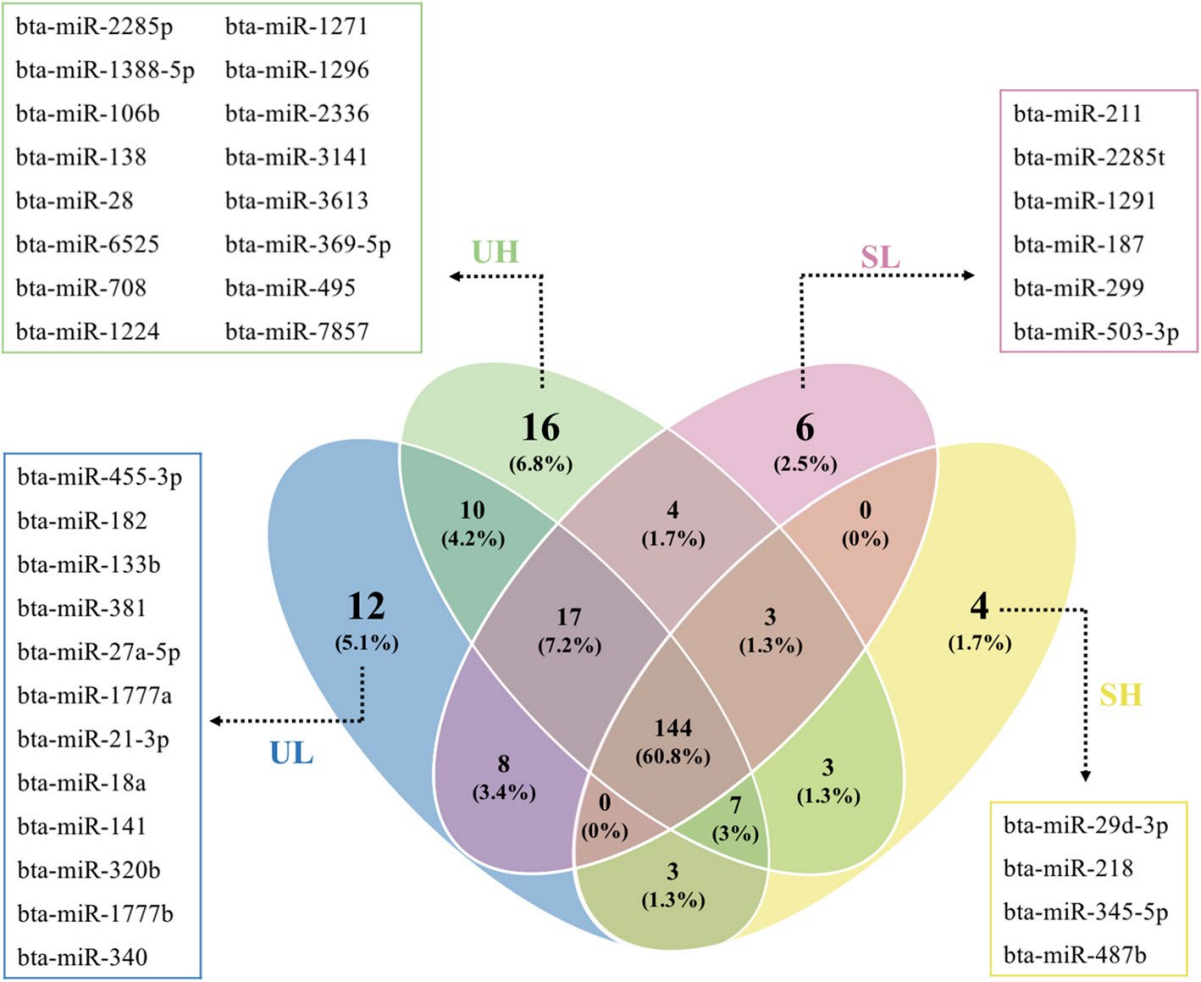
Venn diagram of commonly and uniquely expressed microRNAs in all experimental groups. UH, unstimulated high; UL, unstimulated low; SH, superstimulated high; SL, superstimulated low.

A total of 12 miRNA libraries were prepared from EVs recovered from the blood plasma of unstimulated (UH, UL) and stimulated (SH, SL) heifers with an average number of 17 million raw reads per library. After adapter trimming and quality control, an average of 4 million reads per library were retained. An average of 25% of reads were mapped and the average proportion of annotated miRNAs was 6.5% (Supplementary Table S1). Sequencing data analysis revealed a total of 237 known miRNAs across all groups with at least one counted read in a minimum of two libraries of each group. Moreover, 144 miRNAs were commonly detected in all experimental groups and the highest number of uniquely expressed miRNAs (16 miRNAs) was detected in the UH group (Fig. 3). Members of the let-7 family (including let-7a-5p, let-7b, let-7c and let7f.), miR-126-3p, miR-486, and miR-423-5p were among the most abundant miRNAs in all groups (Table 2). A complete list of all expressed miRNAs in all samples is indicated as raw read counts in Supplementary Table S2.

**Table 2:** List of top 20 most abundant miRNAs from plasma extracellular vesicles obtained from high (H) or low (L) responding heifers before (U) or after superstimulation (S).

Name	Average TPM			
	UL	UH	SL	SH
bta-let-7b	49,217	60,649	49,458	66,564
bta-let-7a-5p	27,182	33,436	24,922	27,713
bta-miR-126-3p	25,704	41,492	21,339	16,185
bta-let-7f.	10,820	13,126	9,646	9,382
bta-miR-486	8,354	10,474	8,495	27,291
bta-miR-423-5p	7,779	7,790	5,740	8,514
bta-miR-150	6,066	6,206	8,736	5,295
bta-let-7c	5,754	6,565	8,980	20,508
bta-miR-342	4,406	5,612	5,530	4,960
bta-miR-1468	3,994	5,113	2,815	2,260
bta-miR-16b	3,801	4,539	3,188	3,893
bta-miR-126-5p	3,677	5,352	3,148	2,118
bta-miR-125a	2,860	3,551	2,489	2,371
bta-let-7i	2,620	3,855	3,181	5,263
bta-miR-223	2,438	4,286	3,596	4,346
bta-miR-125b	2,369	2,675	2,991	5,916
bta-miR-30d	2,325	3,000	2,732	2,311
bta-miR-26a	2,061	2,753	2,141	2,088
bta-miR-191	1,769	2,090	1,730	2,312
bta-miR-21-5p	1,666	2,418	1,975	2,225

TPM: Tags Per Million mapped reads.

### Differential expression analysis

**Figure 4:**
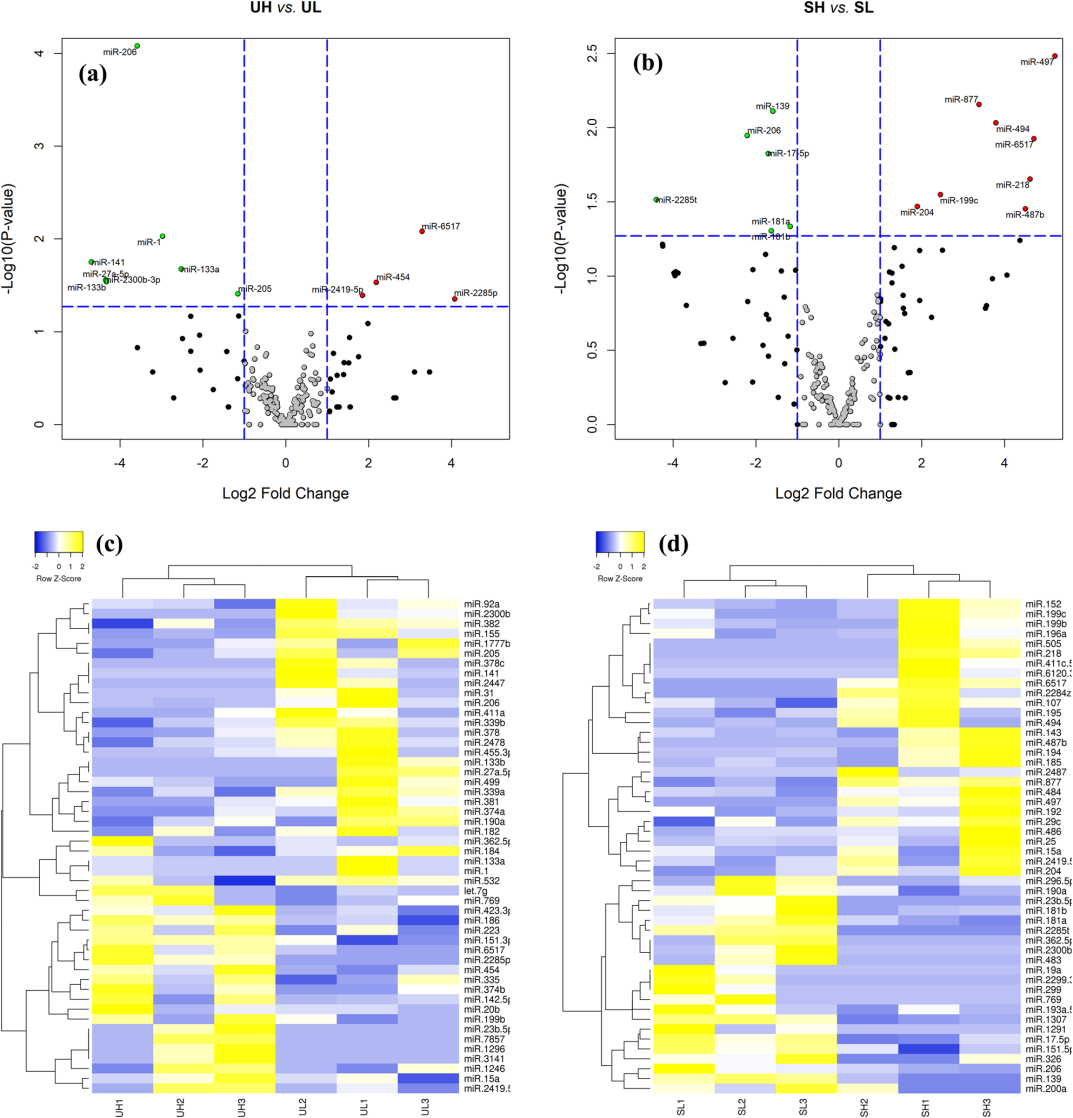
Volcano plot representing microRNA (miRNA) expression level in UH *vs.* UL (**a**) and in SH *vs.* SL heifers (**b**). Each dot represents one miRNA. Upregulated and downregulated miRNAs are labeled with red and green points, respectively. Plots created with R software^88^. Heat map and hierarchical clustering of differentially expressed miRNAs in UH *vs.* UL (**c**) and in SH *vs.* SL heifers (**d**). Yellow and blue colors represent upregulated and downregulated miRNAs, respectively. UH, unstimulated high; UL, unstimulated low; SH, superstimulated high; SL, superstimulated low.

**Figure 5:**
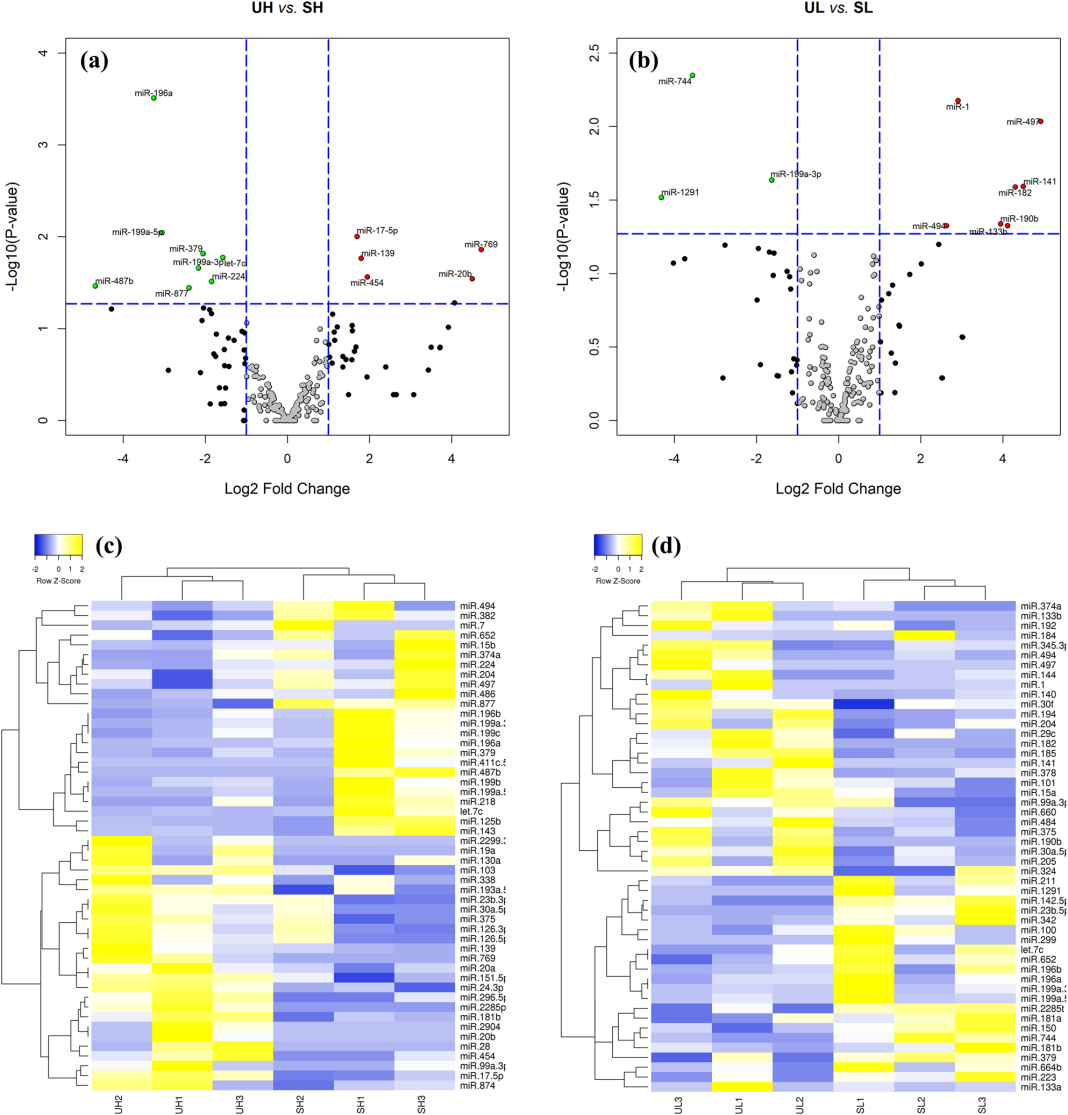
Volcano plot representing microRNA (miRNA) expression level in UH *vs.* SH (**a**) and in UL *vs.* SL heifers (**b**). Each dot represents one miRNA. Upregulated and downregulated miRNAs are labeled with red and green points, respectively. Plots created with R software^88^. Heat map and hierarchical clustering of differentially expressed miRNAs in UH *vs.* SH (**c**) and in UL *vs.* SL heifers (**d**). Yellow and blue colors represent upregulated and downregulated miRNAs, respectively. UH, unstimulated high; SH, superstimulated high; UL, unstimulated low; SL, superstimulated low.

Differential expression analysis of EV-miRNAs from the blood plasma of heifers with a divergent response to superstimulation revealed that 12 and 14 miRNAs were significantly dysregulated in unstimulated (UH *vs.* UL) and stimulated (SH *vs.* SL) heifers, respectively. UH heifers exhibited eight down-regulated (including miR-206, miR-1, miR-141, and miR-133a) and four up-regulated miRNAs (miR-6517, miR-454, miR-2419-5p, and miR-228-5p) compared with UL heifers (Fig. 4a,c). Comparing the same two heifer groups after superstimulation, six down-regulated (including miR-139, miR-206, and miR-17-5p) and eight up-regulated miRNAs (including miR-497, miR-877, miR-494, and miR-6517) were DE in SH compared to SL heifers (Fig. 4b,d). On the other hand, analysis of EV-miRNAs from the same response group of heifers before or after superstimulation revealed that 13 and 10 miRNAs were significantly dysregulated in high (UH *vs.* SH) and low (UL *vs.* SL) responding heifers, respectively. Samples of UH heifers exhibited eight down- (including miR-196a, miR-199a-5p, miR-379, let-7c, and miR-199a-3p) and five up-regulated miRNAs (including miR-17-5p, miR-769, and miR-139) compared to their corresponding samples after stimulation (SH group; Fig. 5a,c). However, samples of UL heifers exhibited three down-regulated (miR-744, miR-199a-3p, and miR-1291) and seven up-regulated miRNAs (including miR-1, miR-497, miR-141, and miR-182) compared to their corresponding samples after stimulation (SL group; Fig. 5b,d).

**Figure 6:**
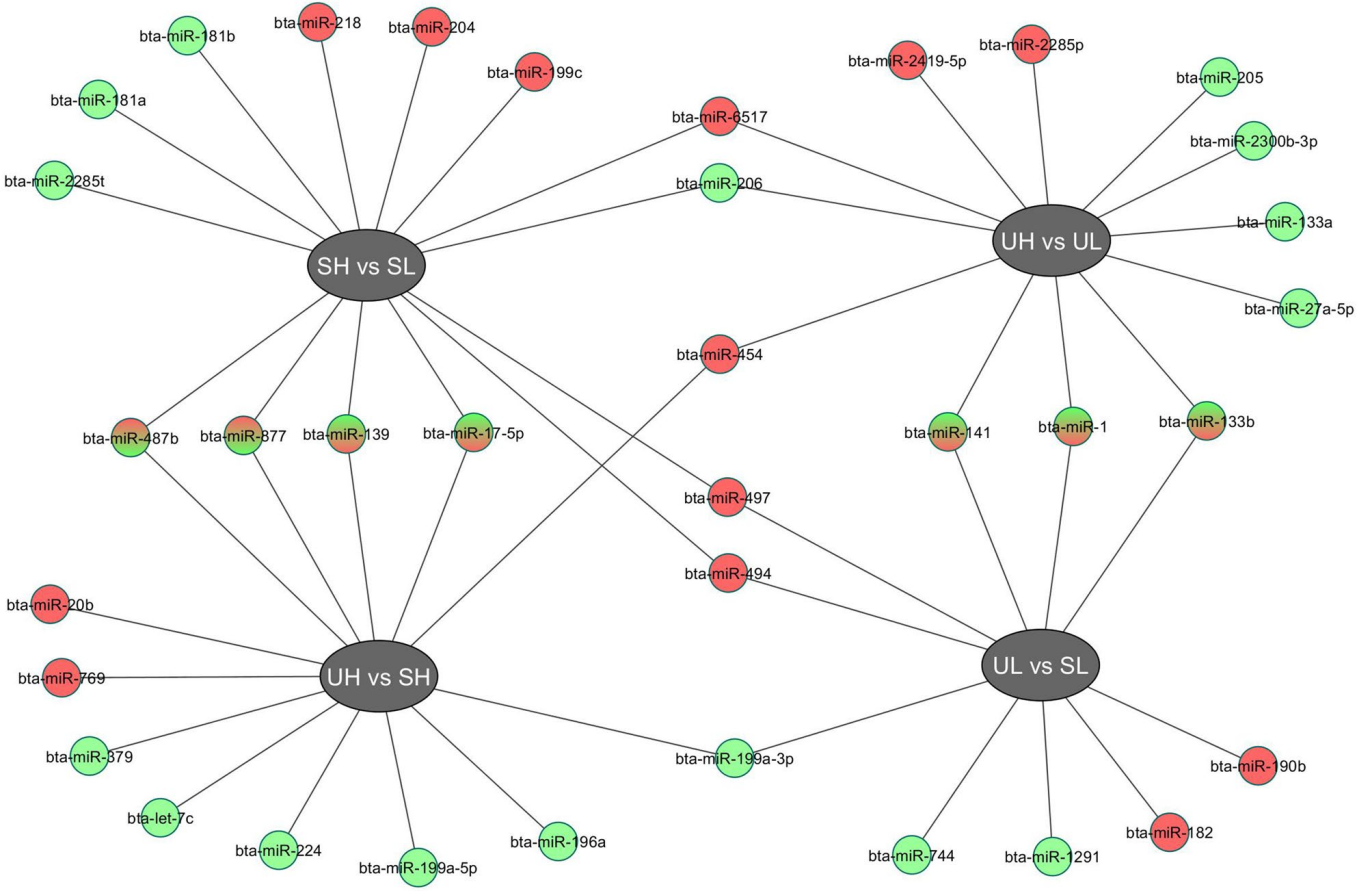
All differentially expressed microRNAs from all comparisons. Upregulated and downregulated microRNAs for each comparison are labeled with red and green, respectively.

Interestingly, comparison of H and L responding U and S heifers revealed two common DE miRNAs (miR-206 and miR-6517) with the same differential expression pattern without or with stimulation. Similarly, miR-199a-3p was commonly DE in U compared to S heifers irrespective of response to superstimulation with the same expression pattern. All DE miRNAs in all comparisons are presented in Table 3 and Fig. 6.

**Table 3:** Differentially expressed (DE) miRNAs from plasma extracellular vesicles obtained from high (H) or low (L) responding heifers before (U) or after superstimulation (S).

UH *vs.* UL			SH *vs.* SL		
MicroRNA	Log_2_ FC	*P-*value	MicroRNA	Log_2_ FC	*P-*value
bta-miR-228-5p	4.1	0.044	bta-miR-497	5.2	3.20E−03
bta-miR-6517	3.3	8.20E−03	bta-miR-6517	4.7	0.012
bta-miR-454	2.2	0.029	bta-miR-218	4.6	0.022
bta-miR-2419-5p	1.8	0.041	bta-miR-487b	4.5	0.035
bta-miR-205	− 1.2	0.039	bta-miR-494	3.8	9.20E−03
bta-miR-133a	− 2.5	0.021	bta-miR-877	3.4	6.90E−03
bta-miR-1	− 3.0	9.30E−03	bta-miR-199c	2.4	0.028
bta-miR-206	− 3.6	8.30E−05	bta-miR-204	1.9	0.034
bta-miR-27a-5p	− 4.3	0.028	bta-miR-181a	− 1.2	0.046
bta-miR-133b	− 4.3	0.029	bta-miR-139	− 1.6	7.70E−03
bta-miR-2300b-3p	− 4.4	0.028	bta-miR-181b	− 1.6	0.049
bta-miR-141	− 4.7	0.018	bta-miR-17-5p	− 1.7	0.015
			bta-miR-206	− 2.2	0.011
			bta-miR-2285t	− 4.4	0.03

UH *vs.* SH			UL *vs.* SL		
MicroRNA	Log_2_ FC	*P-*value	MicroRNA	Log_2_ FC	*P-*value
bta-miR-769	4.7	0.013	bta-miR-497	4.9	9.20E−03
bta-miR-20b	4.5	0.028	bta-miR-141	4.5	0.026
bta-miR-454	1.9	0.027	bta-miR-182	4.3	0.026
bta-miR-139	1.8	0.017	bta-miR-133b	4.1	0.047
bta-miR-17-5p	1.7	9.89E−03	bta-miR-190b	3.9	0.046
bta-let-7c	− 1.6	0.016	bta-miR-1	2.9	6.66E−03
bta-miR-224	− 1.9	0.03	bta-miR-494	2.6	0.047
bta-miR-379	− 2.1	0.015	bta-miR-199a-3p	− 1.6	0.023
bta-miR-199a-3p	− 2.2	0.021	bta-miR-744	− 3.6	4.48E−03
bta-miR-877	− 2.4	0.035	bta-miR-1291	− 4.3	0.03
bta-miR-199a-5p	− 3.1	8.96E−03			
bta-miR-196a	− 3.3	3.08E−04			
bta-miR-487b	− 4.7	0.034

### Clusters and families of differentially expressed miRNAs

A cluster of miRNAs is a set of two or more miRNAs that are transcribed from adjacent genes on the same chromosome (within ~ 10 Kb distance). The exact genomic location and the cluster information for all DE miRNAs were retrieved from the mirBase database. A total of eight DE miRNAs were involved in four different clusters with two miRNAs per cluster (Table 4). In the first cluster, miR-181a and miR-181b were differentially regulated in only one comparison (SH *vs.* SL), both exhibiting the same expression pattern. The other three clusters were involved in different comparisons. In cluster 3 (miR-206 and miR-133b), both miRNAs were down-regulated in the UH compared to the UL group. In addition, miR-133b was up-regulated in UL compared to SL heifers and miR-206 was down-regulated in SH compared to SL heifers. The same pattern was observed in cluster 4 including miR-1 and miR-133a. These expression patterns indicated that these miRNA clusters tend to be less abundant in H *vs.* L responding heifers. On the other hand, cluster 2 (miR-494 and miR-379) showed a higher abundance in SH compared to either SL or UH group (Table 4).

**Table 4:** Differentially expressed miRNAs clusters and their distribution on bovine chromosomes.

Cluster	Comparison	miRNA	Chr	Start	End	Strand	Log_2_ FC	*P-*value
1	SH *vs.* SL	bta-miR-181a	11	95,966,066	95,966,175	+	− 1.2	0.046
	SH *vs.* SL	bta-miR-181b	11	95,967,281	95,967,369	+	− 1.6	0.049
2	SH *vs.* SL	bta-miR-494	21	67,848,049	67,848,133	+	3.8	0.009
	UL *vs.* SL	bta-miR-494	21	67,848,049	67,848,133	+	2.6	0.047
	UH *vs.* SH	bta-miR-379	21	67,840,242	67,840,327	+	− 2.1	0.015
3	SH *vs.* SL	bta-miR-206	23	24,353,667	24,353,752	+	− 2.2	0.011
	UH *vs.* UL	bta-miR-206	23	24,353,667	24,353,752	+	− 3.6	8.33E−05
	UH *vs.* UL	bta-miR-133b	23	24,357,763	24,357,846	+	− 4.3	0.028
	UL *vs.* SL	bta-miR-133b	23	24,357,763	24,357,846	+	4.1	0.047
4	UH *vs.* UL	bta-miR-1	24	34,928,847	34,928,931	+	− 2.9	0.009
	UL *vs.* SL	bta-miR-1	24	34,928,847	34,928,931	+	2.9	0.006
	UH *vs.* UL	bta-miR-133a	24	34,932,166	34,932,252	+	− 2.5	0.021

A miRNA family consists of a group of miRNAs that share a common sequence or structure configuration and subsequently have similar biological functions. As indicated in Table 5, 14 DE miRNAs were involved in seven different families with two miRNAs per family. For example, the miRNA families 29 (miR-133a and miR-133b) and 38 (miR-206 and miR-1) exhibited a common pattern of lower abundance in H compared to L heifers. However, family 18, including miR-494 and miR-487b, exhibited a pattern of higher abundance in the SH compared to the SL group (Table 5).

**Table 5:** Differentially expressed miRNA families and their distribution on bovine chromosomes.

Accession	Comparison	miRNA	Chr	Start	End	Strand	Log_2_ FC	*P-*value
MIPF0000001	SH *vs.* SL	bta-miR-17-5p	12	66,421,236	66,421,319	+	− 1.7	0.014
	UH *vs.* SH	bta-miR-17-5p	12	66,421,236	66,421,319	+	1.7	0.009
	UH *vs.* SH	bta-miR-20b	X	18,021,463	18,021,531	−	4.5	0.028
MIPF0000007	SH *vs.* SL	bta-miR-181a	11	95,966,066	95,966,175	+	− 1.2	0.046
	SH *vs.* SL	bta-miR-181b	11	95,967,281	95,967,369	+	− 1.6	0.049
MIPF0000018	SH *vs.* SL	bta-miR-494	21	67,848,049	67,848,133	+	3.8	0.009
	UL *vs.* SL	bta-miR-494	21	67,848,049	67,848,133	+	2.6	0.047
	SH *vs.* SL	bta-miR-487b	21	67,861,931	67,862,014	+	4.5	0.035
	UH *vs.* SH	bta-miR-487b	21	67,861,931	67,862,014	+	− 4.7	0.034
MIPF0000029	UH *vs.* UL	bta-miR-133b	23	24,357,763	24,357,846	+	− 4.3	0.028
	UL *vs.* SL	bta-miR-133b	23	24,357,763	24,357,846	+	4.1	0.047
	UH *vs.* UL	bta-miR-133a	24	34,932,166	34,932,252	+	− 2.5	0.021
MIPF0000038	SH *vs.* SL	bta-miR-206	23	24,353,667	24,353,752	+	− 2.2	0.011
	UH *vs.* UL	bta-miR-206	23	24,353,667	24,353,752	+	− 3.6	8.33E−05
	UH *vs.* UL	bta-miR-1	24	34,928,847	34,928,931	+	− 2.9	0.009
	UL *vs.* SL	bta-miR-1	24	34,928,847	34,928,931	+	2.9	0.006
MIPF0000040	UH *vs.* SH	bta-miR-199a-5p	16	40,646,156	40,646,259	−	− 3.1	0.008
	UH *vs.* SH	bta-miR-199a-3p	16	40,646,156	40,646,259	−	− 2.2	0.021
	UL *vs.* SL	bta-miR-199a-3p	16	40,646,156	40,646,259	−	− 1.6	0.023
MIPF0000747	UH *vs.* UL	bta-miR-2285p	13	36,776,800	36,776,880	+	4.1	0.044
	SH *vs.*SL	bta-miR-2285t	3	7,976,721	7,976,803	−	− 4.4	0.030

### Novel and putative miRNA expression

A list of novel and putative miRNAs is presented in Supplementary Table S3. One novel miRNA was detected on chromosome 17 the sequence of which matched human hsa-miR-203a. This miRNA was significantly up-regulated in the UL compared to the SL group. A total of 18 putative miRNAs were predicted from the unannotated sequence reads based on the surrounding genome sequence and the structural capability to fold into a hairpin shape. Interestingly, seven out of the 18 putative miRNAs were mapped to an adjacent genome region (~ 10 Kb) on chromosome 25 as one cluster. In addition, two putative miRNAs were significantly up-regulated in SH compared to SL heifers (Supplementary Table S3).

### Target gene prediction, gene ontology, and pathway analysis

Comparing the UH *vs.* UL group, a total of 2363 and 1421 genes were predicted to be targeted by the down- and up-regulated miRNAs, respectively. KEGG pathway analysis revealed that signaling pathways including Hippo, PI3K-Akt, Wnt, Ras, RAP1, and MAPK were the top pathways targeted by the down-regulated miRNAs (Supplementary Fig. S1, Table S4). The PI3K-Akt and RAP1 signaling pathways were also targeted by the up-regulated miRNAs in addition to VEGF signaling, regulation of lipolysis in adipocytes, regulation of cytoskeleton and focal adhesion pathways (Supplementary Fig. S1, Table S4). Among the predicted genes, 225 and 40 were commonly targeted by at least two of the down- and up-regulated miRNAs, respectively (Supplementary Table S5).

Comparison of the SH *vs.* SL groups revealed a total of 1138 and 2224 genes as predictably targeted by the down- and up-regulated miRNAs, respectively. Signaling pathways including MAPK, estrogen (E2), oxytocin, and Wnt in addition to circadian rhythm were the top targeted pathways by the down-regulated miRNAs. However, PI3K-Akt, RAP1, Hippo signaling and focal adhesion pathways were the top targeted pathways by the up-regulated miRNAs (Supplementary Fig. S1, Table S6). Among the predicted genes, 44 and 164 were commonly targeted by at least two of the down- and up-regulated miRNAs, respectively (Supplementary Table S7).

**Figure 7:**
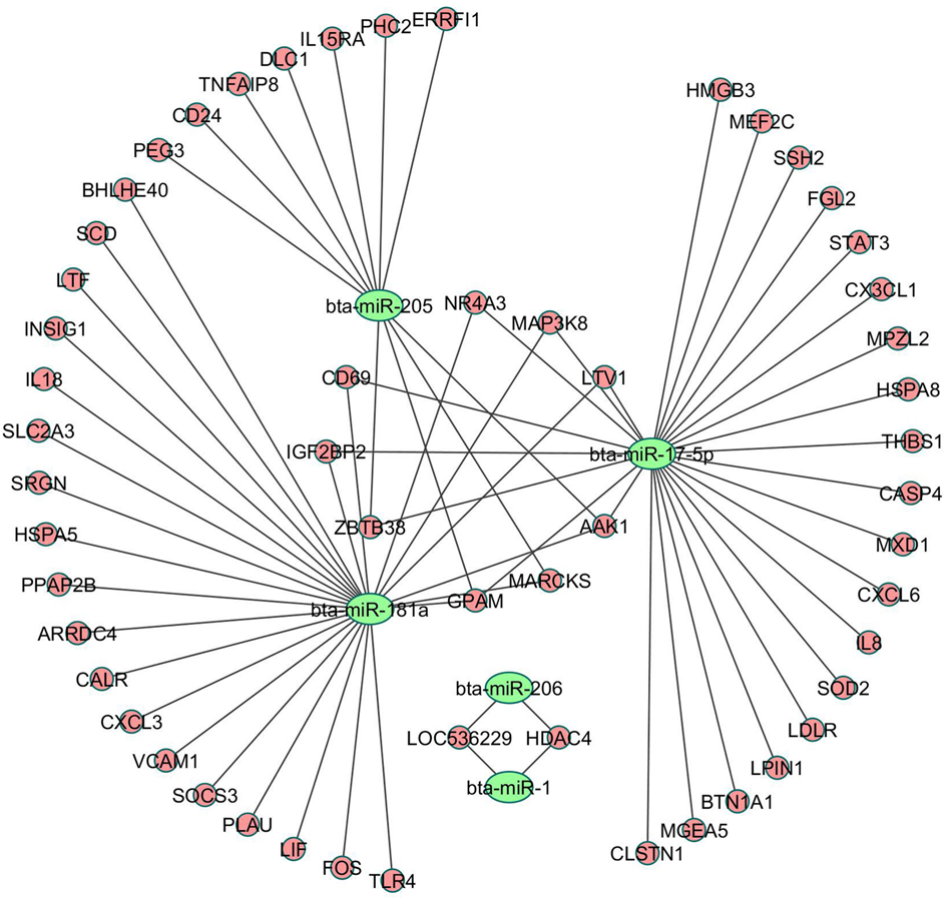
Interaction networking of five down-regulated miRNAs in H compared to L responding heifers and their experimentally validated target genes obtained from miRTarBase 8.0 database.

Five down-regulated miRNAs in H compared to L responding heifers were among the experimentally validated bovine miRNA-target gene list. Interaction networking between these miRNAs and their validated targets is presented in Fig. 7.

### Quantitative reverse transcription PCR (RT-qPCR) validation

**Figure 8:**
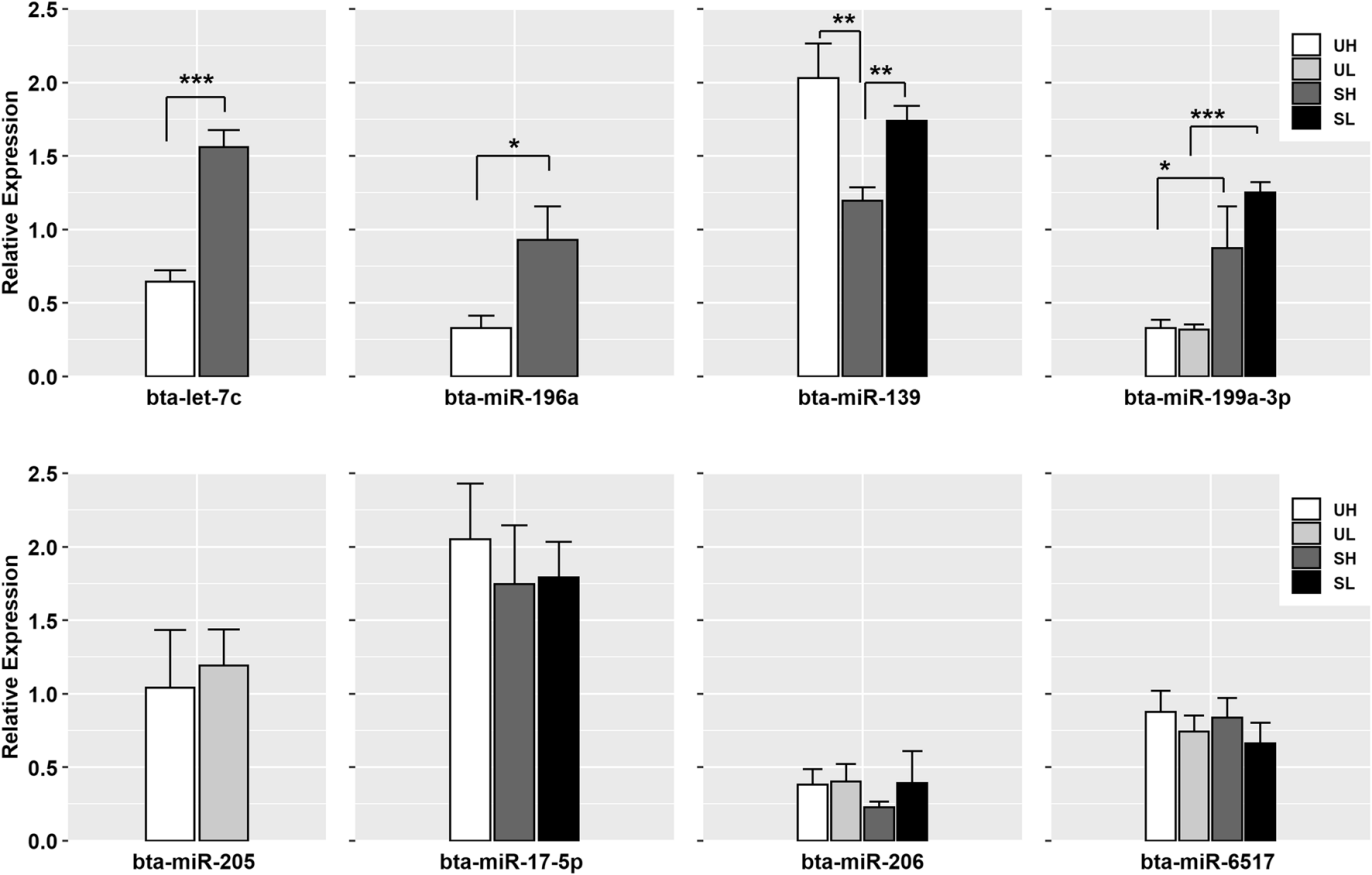
RT-qPCR validation of selected DE-miRNAs in the different group comparisons. Data are shown as means ± SEM (n = 4/group). *P < 0.05, **P < 0.01, ***P < 0.001. UH, unstimulated high; UL, unstimulated low; SH, superstimulated high; SL, superstimulated low. Figure created with R software^88^.

Of the eight DE-miRNAs selected for validation using independent samples and including 13 different comparisons between the groups, four miRNAs including six different comparisons were significantly different, consistent with the RNA-Seq data (*P* < 0.05). The remaining four miRNAs exhibited the same pattern of expression as seen in the RNA-Seq data, but did not reach statistical significance (Fig. 8).

## Discussion

The present study aimed to characterize the plasma EV-miRNA profiles of the same heifers in an U and S cycle taking into account their ovarian response (high or low) to superstimulation treatment. The main findings were that (i) heifers exhibited a significant variation in response to ovarian stimulation, (ii) EV-miRNA profiles in plasma differed between U and S cycles, and (iii) EV-miRNA profiles differed between H and L responding heifers. Data were validated in an independent group of heifers with divergent responses to superstimulation using RT-qPCR. These characterized miRNAs could be useful as biomarkers to predict the superstimulation response of heifers as well as to understand the potential role of miRNAs in follicular development after superstimulation.

In this study, we observed a low mapping rate, and a low proportion of mapped reads were annotated to known miRNAs. This has been commonly observed in the exosomal and extracellular studies compared to cellular RNAseq. For instance, small RNAseq analysis from bovine plasma and blood cells exhibited mapping rates of 38.1% and 94.3%, respectively^40^. Moreover, comparing cellular and exosomal RNA biotypes revealed that exosomes contained a greater proportion of Piwi-interacting RNA (piRNA) and ribosomal RNA, while cells expressed proportionally more miRNA and small nucleolar RNA^41^. In humans, higher percentages of unmapped reads (30–50%) and short reads (20–50%) with a lower proportion of reads annotated to miRNA (2.3–7%) were reported after serum EV small RNAseq analysis^42^. In another study, plasma EV small RNAseq revealed a mapping rate of 27–43% and 3.7% of reads annotated to miRNAs^43^. It has been suggested that the mapping failure of EV-RNAs reads to the reference genome is due to the possibility that EVs may contain genetic material of different sources like microorganisms or other species^44^.

The term EV describes heterogeneous mixtures of vesicles including exosomes and microvesicles^45^. These vesicles are lipid bilayer membrane-enveloped particles containing protein, lipid, RNA, and DNA cargoes and are secreted by almost all cell types into the surrounding microenvironment and body fluids with an emerging role in cellular crosstalk^14,46^. In the last few years, EV-miRNAs have been considered as potential biomarkers for diagnosis and prediction of different physiological and pathological conditions in mammals. For instance, the expression pattern of EV-miRNAs from follicular fluid has been reported to be influenced by energy balance status in postpartum dairy cows^47^ and by body mass index in humans^48^. In relation to ovarian stimulation, distinct miRNA profiles have been identified in serum as predictive molecular markers for ovarian response to stimulation in humans^49^. In cattle, alterations in the abundance of EV-miRNAs in blood plasma and follicular fluid have been correlated with ovarian superstimulation^26^. In our study, potential EV-enclosed miRNA biomarkers of the response to ovarian stimulation were identified in the blood plasma of heifers before and after superstimulation. Comparing H and L responding heifers before (U) and after (S) superstimulation revealed two common DE miRNAs (miR-206 and miR-6517) with the same expression pattern in both statuses. MiR-206 was down-regulated while miR-6517 was upregulated in H compared to L heifers. Previously, it was demonstrated that miR-206 directly targets and reduces endogenous expression levels of E2 receptor-α (ERα) mRNA and protein in human cells^50^. ERα mainly mediates the biological activity and proliferative effects of E2 on the reproductive tissues including the ovarian follicular cells^51^. ERα knockout mouse females were found to be sterile and the vast majority of the preovulatory follicles did not ovulate upon superovulation treatment^52^. These observations were confirmed in ERα knock-in mice, which have a mutation in ERα that disrupts DNA binding, and do not respond to superovulation treatment^53^. A recent study in goats reported that miR-206 expression was down-regulated in the large follicles of multiparous compared to uniparous goats suggesting its potential involvement in the regulation of ovulation rate^54^. Moreover, in the study of Noferesti et al.^26^, miR-206 was down-regulated in the follicular fluid of S compared to U heifers. Based on the mirBase database, we identified four different clusters of miRNAs including two DE miRNAs each (Table 4). In the third and fourth clusters, miR-206, miR-133b, miR-1 and miR-133a were all down-regulated in UH compared to UL heifers. Meanwhile, miR-206 together with miR-1 and miR-133a with miR-133b can be categorized as two miRNA families and therefore are predicted to target the same genes. As described above, miR-206 is inversely correlated with E2 biological activity by targeting ERα. Similarly, miR-133a has been reported to be highly expressed miRNA in the case of E2 deficiency^55^. In contrast, miR-133b was found to stimulate ovarian estradiol synthesis by targeting *Foxl2* mRNA^56^. Several studies identified the three miRNAs, miR-206, miR-1, and miR-133a as muscle-abundant miRNAs and their abundance in serum was considered a biomarker of muscular dystrophy in animal models^57,58^ and human patients^59^. In humans, a strong correlation between muscular dystrophy and diminished ovarian reserve with a low response to ovarian stimulation has been reported^60,61^. These results together with the findings of the current study suggest that miR-206, miR-1 and miR-133a could be potential biomarkers of the ovarian response to superstimulation. Another interesting miRNA family and cluster, including miR-181a and miR-181b, exhibited significant down-regulation in SH compared to SL heifers. Previously, Zhang et al.^62^ demonstrated that miR-181a suppresses the proliferation of mouse granulosa cells via targeting activin receptor IIA mRNA and its expression was reduced in preantral follicles compared to primary follicles. In cattle, the expression of miR-181 family was down-regulated in granulosa cells of subordinate compared to the dominant follicles at day 7 of the estrous cycle^63^. These results indicate a regulatory role of the miR-181 family in follicular recruitment and development and thus in the ovarian response to superstimulation.

Irrespective of the DE miRNAs, we found a number of highly abundant EV-miRNAs in all tested samples (Table 2) with no expression differences among the different comparisons except for let-7c. Members of the let-7 family, including let-7b, -7a-5p, -7c, and -7i, as well as miR-125, miR-126, miR-16b, miR-21-5p, and miR-26a were among the top 20 abundant miRNAs in all samples. All of these miRNAs have been reported among the most abundant miRNAs in whole ovaries or follicular/luteal tissues of several mammalian species (reviewed by Donadeu et al.^64^). In addition, previous studies reported the let-7 family as a highly abundant miRNA family in bovine ovarian and follicular cells^65–67^ as well as throughout the lifespan of the bovine CL^68^ with a significant potential role in ovarian function. .

Plasma EV-miRNA profiles exhibited some differences between U and S cycles in both H and L responding heifers. The effect of superstimulation treatment on the expression level of specific miRNAs correlated with follicular health and development has been reported in the granulosa cells of the stimulated cows^69^. Interestingly, in the current study, we found that miR-199a-3p was commonly up-regulated after superstimulation in both H and L responding heifers. In addition, miR-199a-5p, a member of the same family, showed the same pattern in H responding heifers. Previously, it has been reported that miR-199a family was up-regulated in ovarian granulosa cells of patients with exhibiting a hyper-response to ovarian stimulation compared with normal responding patients^70^. A dysregulation in the expression of miR-199a-3p has been reported in the follicular and luteal phases in the ovine ovary^71^ and in subordinate and dominant follicles at day 7 of the estrous cycle in cattle^63^. In addition, miR-199a was categorized among the most abundant miRNAs in whole ovaries with a potential role in follicular and luteal development^64^. On the other hand, miR-17-5p and miR-182 were down-regulated in the S cycle of H and L heifers, respectively compared to the U cycle. Both miRNAs play a role in regulating luteal steroidogenesis in bovine luteal tissues^72^ and human follicular cells^73^. Recently, de Ávila et al.^74^ investigated the EV-miRNA content of bovine follicles at different stages of the estrous cycle in association with different follicular fluid P4 concentrations. A group of EV-miRNAs was up-regulated in the follicular fluids of the low compared with the high P4 group. Consistent with that study, we observed miR-769, miR-454 and miR-190b to be up-regulated in the U compared to the S cycle. However, miR-379, miR-196a and miR-487b exhibited an opposite pattern. These results together with our findings give more insights into the potential role of miRNAs in ovarian function during estrous cycle stages and superstimulation.

Pathway analysis revealed that signaling pathways (including Hippo, PI3K-Akt, Wnt, E2, oxytocin, and MAPK) were among the top significant pathways enriched with genes targeted by DE miRNAs in H *vs.* L responding heifers (Supplementary Fig. S1). Signaling pathways play a critical role in regulating ovarian function and development (reviewed by Prasasya & Mayo^75^). For instance, the Hippo and PI3K-Akt signaling pathways are known to regulate follicular recruitment and development. Activation of PI3K-Akt with the disruption of the Hippo signaling pathway together accelerate primordial follicles recruitment^76,77^. Oxytocin and E2 signaling were the top pathways targeted by potential biomarker miRNAs detected in the serum of hyper responding patients to ovarian stimulation^49^. In addition, MAPK, Wnt and PI3K signaling were among the top pathways targeted by dysregulated miRNAs during the growth and selection of dominant follicles in cattle^78^ and by exosomal miRNAs in human follicular fluid during follicular maturation^79^. This highlights the significant role of miRNAs in controlling ovarian function through targeting the different signaling pathways. However, as the mechanisms that regulate the incorporation of specific miRNA into EVs and the uptake by recipient cells are still unknown, caution should be applied while interpreting the potential function of these detected EV-miRNAs. Another interesting pathway was the circadian rhythm pathway (including *CLOCK* gene). It was the top significant pathway enriched with genes targeted by down-regulated miRNAs in SH compared to SL heifers. The circadian rhythm system is controlled by the transcription of circadian clock genes and regulates several physiological reproductive processes in mammals including ovarian function, responsiveness to gonadotropins and ovulation^80^. In cattle, the expression of circadian genes is associated with ovarian follicle development from the recruitment to the ovulatory phase^81^. In addition, the expression level of *CLOCK* mRNA was positively correlated with the production of E2 which was stimulated by FSH in cultured granulosa cells^81^. Moreover, Gräs et al.^82^ demonstrated that the expression of circadian genes becomes rhythmic during gonadotropin-dependent folliculogenesis in the rat ovary, suggesting a functional association between ovarian circadian genes and P4 production in preovulatory/ovulatory follicles and CL. Accumulated evidence indicates that EVs with their non-coding RNA cargo plays an important role in regulating the circadian rhythm of individual cells through a post-transcriptional mechanism^83,84^. Specifically, miR-206 has been reported as a mediator of the dynamic mechanism of circadian rhythm in mammals^85^. In addition, miR-17-5p and miR-181a, which were also down-regulated in SH compared to SL heifers, were considered as important modulators of circadian rhythm related genes^86,87^.

In conclusion, findings indicate that heifers with divergent responses to ovarian superstimulation exhibit differential abundance of plasma EV-miRNAs which may be used as a potential biomarker to predict individual animal response. In addition, our results indicate a potential role of miRNAs in follicular development after superstimulation.

## Supplementary information

Supplementary Figure

Supplementary Tables
