# Supplementary material for: Plasma extracellular vesicle miRNAs as potential biomarkers of superstimulatory response in cattle
Source: Sci Rep. 2020 Nov 5;10:19130. doi: 10.1038/s41598-020-76152-9 (PMC7645755; doi:10.1038/s41598-020-76152-9)
Supplement: Supplementary file 1 — Supplementary Figure [file 41598_2020_76152_MOESM1_ESM.pdf]

Plasma extracellular vesicle miRNAs as potential biomarkers of superstimulatory response in cattle

Ahmed Gad, José María Sánchez , John A. Browne, Lucie Nemcova, Jozef Laurincik, Radek Prochazka, Pat Lonergan

Supplementary Fig. S1: Interaction networking of top pathways enriched in predicted genes targeted by down-regulated (A) and up-regulated (B) microRNAs in UH compared to UL group and by down-regulated (red) and up-regulated (green) microRNAs in SH compared to SL group (c). UH, unstimulated high; UL, unstimulated low; SH, superstimulated high; SL, superstimulated low.

A

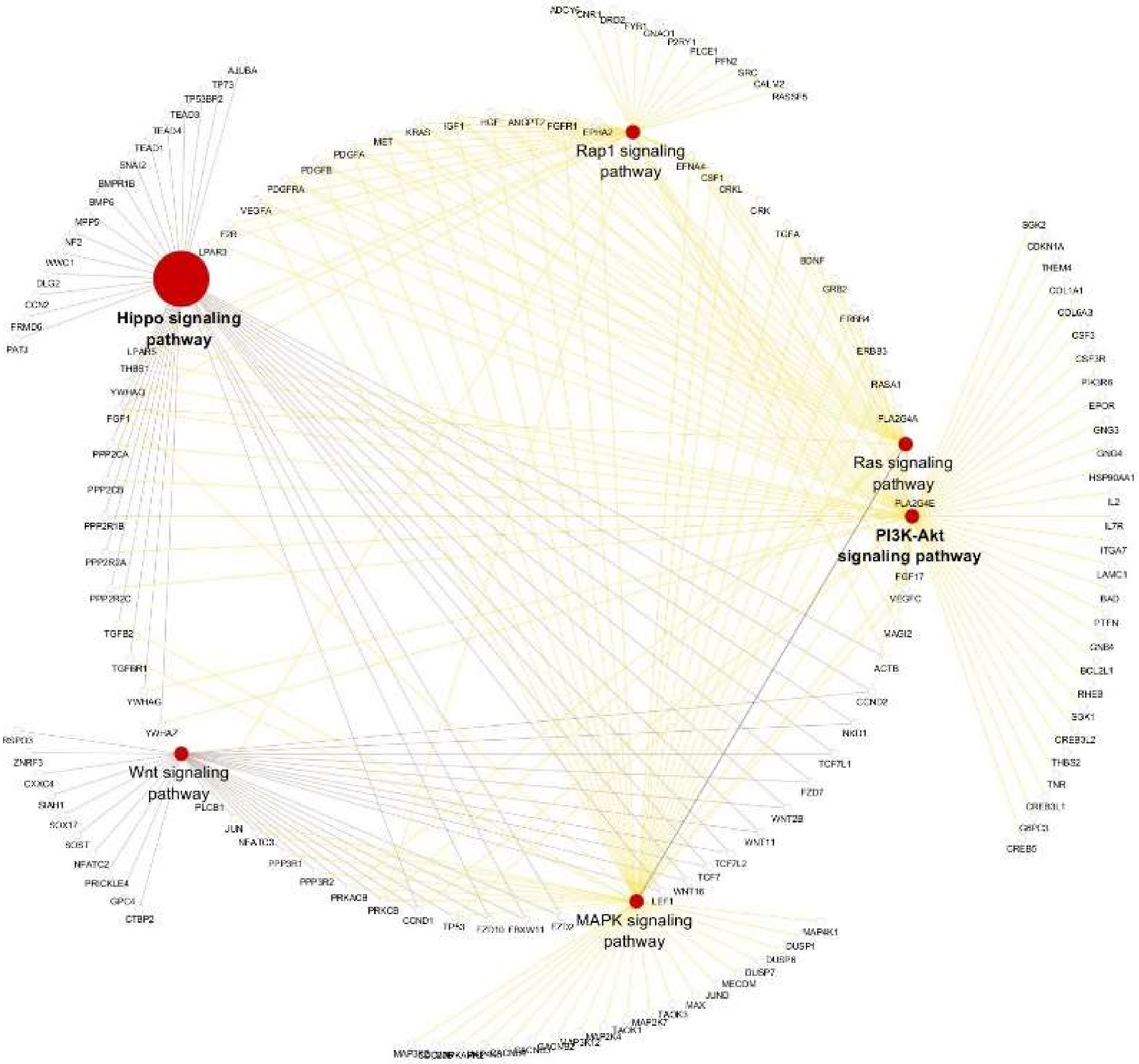

Hippo signaling pathway
Rap1 signaling pathway
Ras signaling pathway
PI3K-Akt signaling pathway
Wnt signaling pathway
MAPK signaling pathway
AJUBA
TP73
TP53BP2
TEAD3
TEAD4
TEAD1
SNAI2
BMPR1B
BMP6
MPP5
NF2
WWC1
DLG2
CCN2
FRMD6
PATJ
LPAR3
LPAR5
THBS1
YWHAQ
FGF1
PPP2CA
PPP2CB
PPP2R1B
PPP2R2A
PPP2R2C
TGFB2
TGFBR1
YWHAG
YWHAZ
F2R
VEGFA
PDGFRA
PDGFB
PDGFA
MET
KRAS
IGF1
HGF
ANGPT2
FGFR1
EFNA2
EFNA4
CSF1
CRKL
CRK
TGFA
BDNF
GRB2
ERBB4
ERBB3
RASA1
PLA2G4A
PLA2G4E
FGF17
VEGFC
MAGI2
ACTB
CCND2
NKD1
TCF7L1
FZD7
WNT2B
WNT11
TCF7L2
TCF7
WNT16
LEF1
ADCY5
CNR1
DRD2
TYR1
GNAO1
P2RY1
PLCE1
PFN2
SRC
CALM2
RASSF5
SGK2
CDKN1A
THEM4
COL1A1
COL6A3
CSF3
CSF3R
PIK3R6
EPOR
GNG3
GNG4
HSP90AA1
IL2
IL7R
ITGA7
LAMC1
BAD
PTEN
GNB4
BCL2L1
RHEB
SGK1
CREB3L2
THBS2
TNR
CREB3L1
G6PC3
CREB5
RSPO3
ZNRF3
CXXC4
SIAH1
SOX17
SOST
NFATC2
PRICKLE4
GPC4
CTBP2
PLCB1
JUN
NFATC3
PPP3R1
PPP3R2
PRKACB
PRKCB
CCND1
TP53
FZD10
FBXW11
FZD2
MAP4K1
DUSP1
DUSP6
DUSP7
MECOM
JUND
MAX
TAOK3
MAP2K7
TAOK1
MAP2K4
MAP3K12

B

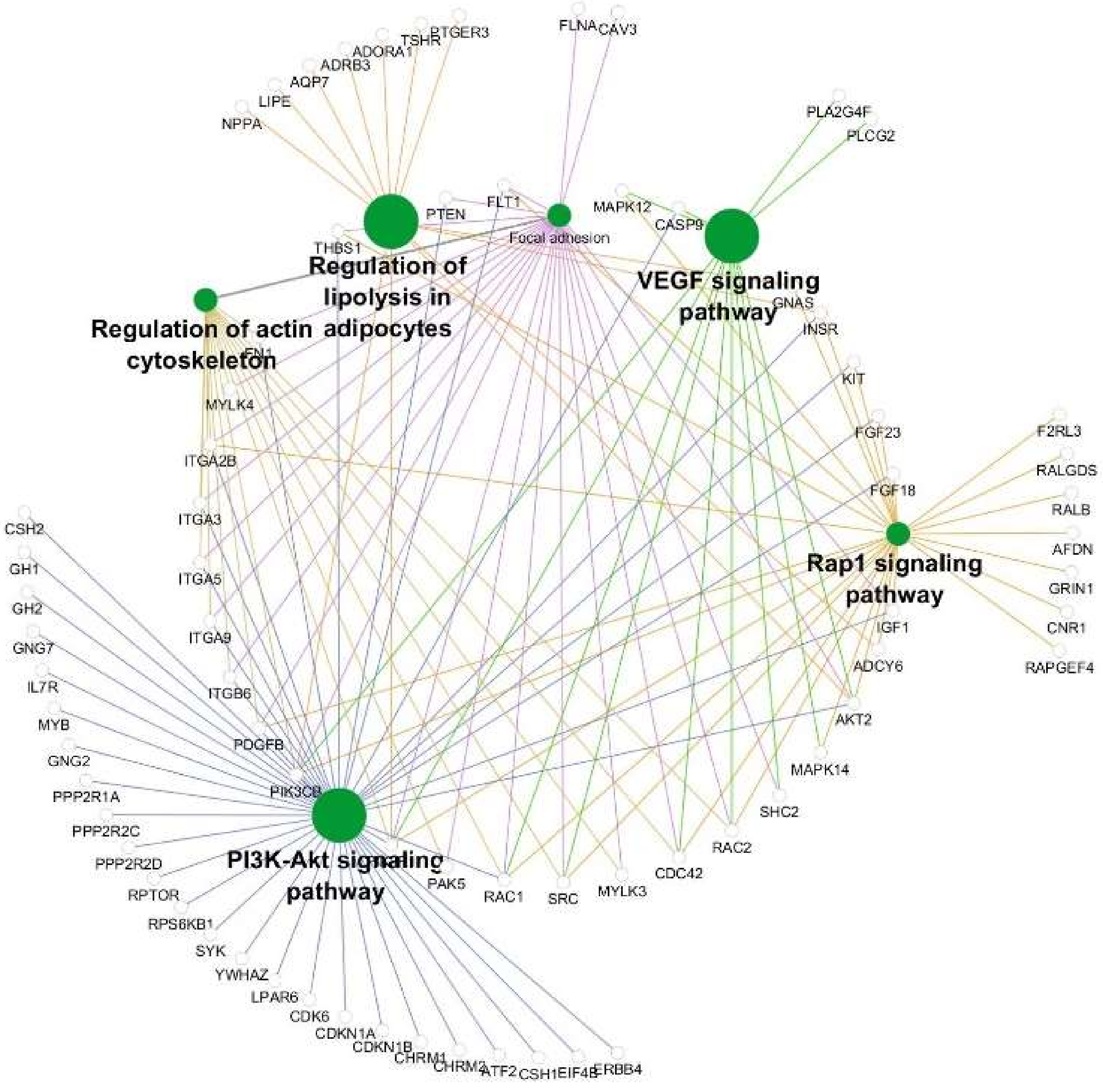
Regulation of lipolysis in adipocytes
Focal adhesion
VEGF signaling pathway
Regulation of actin cytoskeleton
Rap1 signaling pathway
PI3K-Akt signaling pathway
NPPA
LIPE
AQP7
ADRB3
ADORA1
TSHR
PTGER3
FLNA
CAV3
PLA2G4F
PLCG2
FLT1
PTEN
THBS1
MAPK12
CASP9
GNAS
INSR
KIT
FGF23
FGF18
F2RL3
RALGDS
RALB
AFDN
GRIN1
CNR1
RAPGEF4
IGF1
ADCY6
AKT2
MAPK14
SHC2
RAC2
CDC42
MYLK3
SRC
RAC1
PAK5
FN1
MYLK4
ITGA2B
ITGA3
ITGA5
ITGA9
ITGB6
PDGFB
PIK3CB
CSH2
GH1
GH2
GNG7
IL7R
MYB
GNG2
PPP2R1A
PPP2R2C
PPP2R2D
RPTOR
RPS6KB1
SYK
YWHAZ
LPAR6
CDK6
CDKN1A
CDKN1B
CHRM1
CHRM2
ATF2
CSH1
EIF4B
ERBB4

C

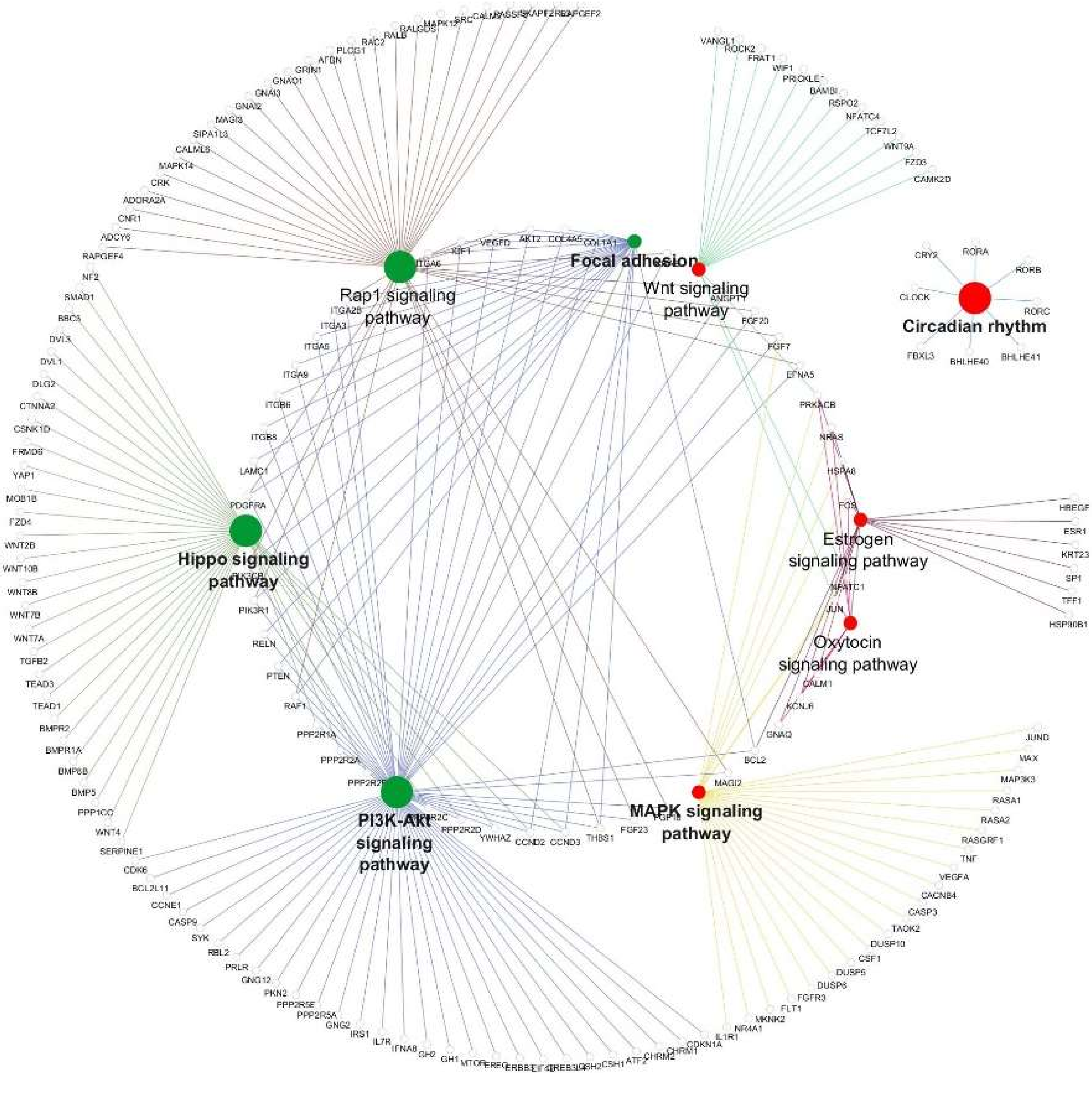
Rap1 signaling pathway
Focal adhesion
Wnt signaling pathway
Circadian rhythm
Hippo signaling pathway
Estrogen signaling pathway
Oxytocin signaling pathway
PI3K-Akt signaling pathway
MAPK signaling pathway
